# Interventions and working relationships of voluntary organisations for diabetes self-management: A cross-national study

**DOI:** 10.1016/j.ijnurstu.2017.02.001

**Published:** 2017-05

**Authors:** Mari Carmen Portillo, Anne Kennedy, Elka Todorova, Elena Regaira, Michel Wensing, Christina Foss, Christos Lionis, Ivaylo Vassilev, Valentin Goev, Anne Rogers

**Affiliations:** a Faculty of Health Sciences, NIHR Wessex CLAHRC, University of Southampton, Hampshire, UK; b Department of Sociology, University of National and World Economy, Sofia, Bulgaria; c Quality Department, University Clinic of Navarra, Pamplona, Spain; d Department of General Practice and Health Services Research, Heidelberg University Hospital, Heidelberg, Germany; e Institute of Health and Society, University of Oslo, Norway; f Clinic of Social and Family Medicine, Faculty of Medicine, University of Crete, Crete, Greece; g Department of Statistics, University of National and World Economy, Sofia, Bulgaria

**Keywords:** Diabetes mellitus, Europe;, Financial crisis, Health services, Self-management, Mixed methods, Voluntary organisations and community groups

## Abstract

**Background:**

Diabetes has become a challenging health priority globally. Given the tensions of financially burdened health systems in Europe the mobilisation of community resources like voluntary organisations and community groups is seen as a health policy strategy to sustain the management of long-term conditions like diabetes. However, little is known about how this is happening in practice in Europe.

**Objectives:**

To explore diabetes self-management interventions undertaken or promoted by voluntary organisations and community groups in Europe; and describe the types of working relationships between these organisations, European health systems and users when implementing diabetes self-management programmes in different areas.

**Design:**

A mixed method study (survey/qualitative interviews) was undertaken. This research formed part of a European project (7th Framework programme of the European Commission) exploring the link between resources, like community organisations, and peoples’ capacities to manage long-term conditions.

**Settings:**

Six European countries (Bulgaria, Greece, Norway, Spain, the Netherlands and the United Kingdom) participated in the study. Three areas: deprived urban area, a relatively affluent urban area and a deprived rural area were purposefully selected.

**Participants:**

Through a purposeful sample and bottom up strategies 749 representatives of voluntary organisations and community groups were recruited from the geographical areas above. Organisations with at least three members, existing for at least one year that could provide information or other type of support directly or indirectly relevant to patients with diabetes were included.

**Methods:**

Participants completed a 15 item questionnaire for the survey (n = 749) and a voice recorded semi structured interview (n = 300). Data collection focused on the type of activities and roles developed to promote health, and relationships and communication channels between organisations, health services and users. Descriptive and comparative statistical and qualitative content analyses were used.

**Results:**

Participants perceived they had better reach of people with health needs than health providers, filled the administration gaps left in their capacity to deal with basic diabetes practical needs, humanized care, and acted as mediators between services and communities. There were significant differences between countries in relation to the types of activities (p-value < 0.001), roles (p-value < 0.001) and funding sources (p-value < 0.001) of organisations concerning diabetes self-management. In non-affluent countries organisations tend to promote social activities twice more often.

**Conclusions:**

Community and voluntary organisations provide complimentary and on-going support in diabetes management. This involves a shift from focusing on the illness to also longing for social cohesion, sense of community and wellbeing in diabetes health practices and policies.

## What is already known about the topic?

• European action advocates alliances between professionals, communities and voluntary organisations for more cost-effective educational interventions when dealing with non-communicable diseases like diabetes.
• The involvement of voluntary organisations and community groups in diabetes self-management programmes has been proved to decrease costs for health systems, improve knowledge and increase self-efficacy and sense of belonging among others.
• Relationships between professionals and voluntary organisations and community groups when planning and implementing diabetes self-management programmes need to be based on principles of assistance, support, sharing and link.

## What this paper adds

• Voluntary organisations and community groups’ involvement in self-management initiatives result in the humanization of services, overcoming a great challenge for self-management policy makers and health professionals.
• Bottom up diabetes self-management initiatives constitute a clear shift from a specific illness focus to holistic complexity, and aspirations for social cohesion and wellbeing, crucial issues nowadays.
• Relationships between health care professionals and voluntary organisations in diabetes self-management initiatives and educational programmes lack acknowledgement and synchronization, shifting organisations from complementary to substitute roles due to action-planning gaps.

## 1 Background

The number of people with diabetes is increasing in both developed and developing countries, affluent and deprived populations, and people of all ages (Hu, 2011, Tabish, 2007, Wild et al., 2004, World Health Organisation, 2016). Concretely, in Europe diabetes has become a challenging health problem, which is growing simultaneously to health inequalities, ageing population or untenable health systems (Scheller-Kreinsen et al., 2009). Health and social policy and systems face difficulties to ensure chronic disease management and measures like integrated care models and self-management programmes are seen as potential solutions (Scheller-Kreinsen et al., 2009).

Traditional didactic education programmes have been developed to manage the biomedical consequences of diabetes and encourage individual self-management to help people adopt healthy behaviours and achieve positive biomedical and lifestyle outcomes (European Commission Health, 2007, Khunti et al., 2012, Tricco et al., 2012). Nevertheless, these approaches do not seem an effective or sustainable solution when taking the life-span of people with long-term conditions like diabetes into consideration (European Commission Health, 2007, Penn et al., 2015) or approaching the complexity of cases nowadays (Nolte et al., 2014). Furthermore, self-management programmes lack an effective mobilisation of community resources to mitigate the serious consequences of chronic conditions like diabetes (such as early death, loss of limbs and sight, and isolation) in times of growing inequalities (Coulter et al., 2008, Khunti et al., 2012, World Health Organisation Regional Office for Europe, 2011). In this sense, integrated models of care have potential to manage complex long-term conditions like diabetes as an attempt to avoid care delivery fragmentation and broaden out the care and support available for people beyond an illness focused approach (Coulter et al., 2008, Shrivastava et al., 2013). This is clearly a priority in the reduction of risk and management of non-communicable diseases in Europe, which highlights that alliances between professionals, communities or voluntary organisations and community groups are part of the action plans for more cost-effective educational interventions (World Health Organisation Regional Office for Europe, 2011). This is vital since day-to-day decisions related to diabetes are mainly taken by individuals in the context of their personal communities of support and own financial and social possibilities (Vassilev et al., 2013).

In contexts with different levels of deprivation community resources gaining increasing salience are voluntary organisations and community groups (Portillo et al., 2015), which seem to offer universal, innovative and person centred alternatives to people in social and health need (South et al., 2012). Also people join community groups often in response to a health-related event and social contact associated with continued participation in voluntary organisations and community groups is viewed as helping with long-term condition management (Jeffries et al., 2015). There is evidence of benefits of the involvement of voluntary organisations and community groups in diabetes self-management and/or other behaviour change programmes such as the decrease in costs for health systems, improvement in knowledge, uptake of healthy behaviours, and increased self-efficacy and emotional support, socialisation and sense of belonging (Collinsworth et al., 2013, Koniak-Griffin et al., 2015, Jeffries et al., 2015, Portillo et al., 2015). The latter can be seen as complementing professional led diabetes self-management programmes shown to decrease health services costs and diabetes complications, and target Ac1 levels and glycaemic stability (Tricco et al., 2012).

Although the contribution to diabetes management of macro (society structures at national or governmental levels), meso (middle groups of organisations like communities, voluntary sector or neighbourhoods) and micro (local individual level e.g. personal networks) societal levels of action (Rogers et al., 2015, Turner, 2016) seems established, the roles and forms of collaboration in self-management programmes when these sectors converge remain ambiguous, challenging the aspirations for alliances expressed in health policy documents (Haas et al., 2014, World Health Organisation Regional Office for Europe, 2011). In this regard, a critical interpretative synthesis of the literature was undertaken (Portillo et al., 2015) showing: 1) the positive impact of voluntary organisations and community groups on the patients education in terms of clarification of doubts, learning outcomes and confidence, 2) the relevance of collaborative plans between lay educators and health care providers to the longer sustainability of the programmes, and 3) the need for professional training of volunteers in self-management programmes. Furthermore, this review suggested that the principles of: assistance (managing daily needs or medication), support (at emotional and social levels), sharing (illness, culture, beliefs) and link (between users, community resources and health professionals) constituted the backbone in the construction of relationships between professionals and voluntary organisations and community groups when planning diabetes self-management programmes at a community level (Portillo et al., 2015). This is in line with what it is transmitted in European Health policies regarding person centred care, understood as compassionate, individualised, holistic, and empowering care for shared decision making (Lusk and Fater, 2013, Morgan and Yoder, 2012) and the need for alliances between all the agents involved in diabetes management (World Health Organisation Regional Office for Europe, 2011). However, little is known about whether macro-meso level action is contemplated when diabetes self-management programmes are implemented in Europe; and about the distribution of responsibilities at macro and meso levels (Powers et al., 2015).

Based on these findings from the literature the following research questions were posed for this study:

1. What diabetes self-management interventions are undertaken or promoted by voluntary organisations and community groups in Europe, and how?
2. Are the principles of assistance, support, sharing and link emerging from the literature integral to those interventions?
3. What are the types of working relationships between voluntary organisations and community groups and European health systems when implementing diabetes self-management programmes with people living in areas with different levels of deprivation?

## 2 Methods

### 2.1 Design

A study with a convergent mixed method design using quantitative and qualitative methods (Creswell and Plano Clark, 2011) was undertaken in 6 European countries (Bulgaria, Greece, Norway, Spain, the Netherlands and the United Kingdom). This research was part of a European project (http: www.eu-wise.com), which aimed to describe and analyze the macro, meso, and micro level strategies that promote the implementation of self-management strategies with people with long-term conditions like diabetes.

The convergent mixed methods design was chosen and both methods were complementary (Creswell and Plano Clark, 2011) because the quantitative data captured the different types of activities, funding sources and other characteristics of the organisations, and the qualitative data helped understand the activities, roles and relationships, and the feelings of participants about their “activities and/or roles” when supporting people with diabetes.

This convergent design involves separate quantitative and qualitative data collections and analyses, and the results are mixed during the interpretation step after both types of data have been collected and analysed independently. More concretely, for this study a *side by side comparison* with separate reports of the quantitative and qualitative findings is presented in the results section and further comparative integration and merging of the two sets findings can be found in the conclusions section (Creswell and Plano Clark, 2011). For the interpretation step we looked at the similarities and differences, relationships and conceptual contribution of the two sets of findings in relation to the research questions.

### 2.2 Ethical approval

Ethical approval was obtained from the pertinent ethics committees in all the countries involved in the project for all data collection stages. No ethical conflicts emerged.

### 2.3 Setting and participants

In each country data collection was completed in October 2013 in three purposefully selected areas (relative to each country): deprived urban area, a relatively affluent urban area and a deprived rural area (Koetsenruijter et al., 2014). Based on a typology of organisations previously developed (Jeffries et al., 2015, Koetsenruijter et al., 2014) (Table 1), we aimed to recruit representatives from a variety of voluntary organisations, operating at local, regional or national levels.

**Table 1:** Types of organisations initially approached and definitions of their focus, and inclusion criteria.

TYPOLOGY	DETAILS	INCLUSION CRITERIA
*Diabetes related organisations*	Groups and organisations with a direct focus on health improvement and especially on diabetes e.g. diabetes foundations and diabetes education groups, associations, forums.	• Organisations that focused on diabetes, health and healthy lifestyle, and wellbeing, and could provide information, treatment, practical help, or other type of support that was directly or indirectly relevant to patients with diabetes. • Organisations of a certain size (more than just 2 or 3 participants/members), although small organisations (e.g. 10 members) could be also included. • Organisations which have existed for at least 1 year.
*Health and healthy lifestyle related organisations*	Groups and organisations with a focus on health outcomes but not immediately focused on people with diabetes. These could include, for example, exercise related organisations or diet groups. Directly/indirectly relevant to diabetes.	
*Wellbeing related organisations*	Community centres where people meet and through which they also socialise, which were directly/indirectly relevant to diabetes. • Organisations for elderly people, which may have impact on self-care behaviours (walking groups, discussion circles). • Sport organisations with activities that might have impact on diabetes people. • Life-style related groups which are set up by professional healthcare providers or other health-related organisation.	
*Patient́s and peoplés rights*	Groups and organisations with a focus on human rights, equality, and fighting discrimination of any type (poverty, disability, gender). Good examples of these organisations could be the Red Cross and its national/regional/local branches or Caritas. Directly/indirectly relevant to diabetes.	
*Other Community groups*	Other less formally groups of people working in the community developing activities for environmental, economic or social good. These could include local neighbourhood centres, hobbies, gastronomy or culture related centres, which could have people with diabetes as members or volunteers.	

### 2.4 Sampling and sample

One or more representatives per organisation were recruited and were given a unique code, indicating the country. A representative was a person involved with the management, day to day operations, and/or the strategic development of the organisation. Applying the inclusion criteria (Table 1), potential organisations and representatives were recruited through a purposeful sampling approach (Palinkas et al., 2015) and the following strategies were adopted in all the participating countries:

1. Literature review/websites searches (Portillo et al., 2015).
2. Previous data collection of the EUWISE project with patients with diabetes, who identified organisations (Koetsenruijter et al., 2014, Koetsenruijter et al., 2016).
3. Previous knowledge of organisations supplemented by a list of organisations suggested by the other project partners (to foster the inclusion of similar organisations across settings) (Koetsenruijter et al., 2014).
4. Snowballing from organisation to organisation.

Furthermore, several team discussions were held about the type of organisations and the sampling frame from which the organisations could be selected during project meetings to ensure appropriate diverse recruitment according to the defined typology (Table 1).

### 2.5 Data collection and analysis

A total of 749 representatives of voluntary organisations and community groups completed a questionnaire with 15 questions relating to contextual information about the organisation, type of activities and roles developed, funding sources and level of operation (Koetsenruijter et al., 2014). The questionnaire was completed in approximately 10 min.

Three hundred out of the 749 participants also completed a semi-structured interview (n = 50 per country). Interviews were carried out by phone or face-to-face in the organisations’ premises. Interviews covered the following issues:

• Organisations’ knowledge about ‘self-management support’, or ‘support for people with Diabetes.
• The contribution of the local/voluntary sector to tackling health inequalities and collaboration between organisations and European health systems.
• The role of each organisation and impact on the health of members with diabetes.

The average length of the semi-structured interviews was 45 min (voice recorded).

Descriptive and comparative statistical tests were carried out (IBM SPSS version 22) and have been detailed under each results table. The significance level was established at p-value < 0.05.

Furthermore, the 300 semi-structured interviews were transcribed verbatim. Due to the large amount of qualitative data a theoretical sampling was applied (Teddlie and Yu, 2007) to select the richest interviews (for this analysis one participant per organisation) that could provide rich information in relation to the research questions of this paper. A total of 78 interviews representing 78 organisations (Bulgaria: n = 10; Greece: n = 10; Norway: n = 10; Netherlands: n = 10; Spain: n = 20; United Kingdom: n = 18) were selected from all the participant countries and were individually and content analysed (Miles et al., 2014).

For the qualitative analysis the following process was completed:

• *First and second level coding*. The *first level coding* started with several readings and the categorisation of the full transcriptions of the selected interviews from Spain (workpackage leader) and UK. This first level coding resulted in 142 descriptive codes. After this, as part of the *second level coding* conceptual connections between first level codes were drawn to create wider and more synthetic subthemes and themes in relation to the research questions.
• To increase the rigour of the process two researchers were involved in these two coding stages, discussing emerging codes and themes and reaching consensus for conceptual and/or language discrepancies between the two countries (Spain-UK) (Hsieh and Shannon, 2002, Miles et al., 2014).
• Finally, a report of the findings from the first and second coding process of the interviews from Spain was written in English and shared with the other participating countries, which constituted an illustrative example-template and unified analysis guidelines to ensure rigour and consistency in the analysis of interviews in the other countries.
• *Further analysis and country comparisons*. The other countries (Bulgaria, Greece, Netherlands and Norway), apart from selecting the most representative interviews and following the template, were instructed to select the richest quotes that could better illustrate the findings in each country for each research question and write a country report. The reports and quotes from all the countries were then read and analysed for country comparisons in line with the research questions of this paper and themes emerging from the coding. Further cross country consultations and discussion took place at this stage to ensure the final cross country report of the findings from the interviews represented the participating countries.
• *Language and translation issues.* The transcriptions of the interviews were analysed in their original language in each country. Then, the reports from each country were written in English-including the quotes. This was decided upon to ensure that the first coding stage clearly represented the data and there was no space for widely divergent interpretations or cultural loss. Also as the researcheŕs language in each country was the same as the participants, this was considered the wisest decision to gain more sensitivity to the raw data (Van Ness et al., 2010).

Moreover, although members of the teams in each country were competent in English, cross country consultation and discussions took place to not only prevent language misunderstandings or interpretations from influencing the reporting of the findings but also contemplate conceptual and context related issues emerging from the analysis that were less rooted in accurate translation of verbatim language (Temple and Young, 2004).

## 3 Results

### 3.1 Survey main results

Table 2, Table 3 show characteristics of the representatives and organisations participating in the survey. Representatives had an average experience working in the voluntary organisations and community groups of 9.1 years with significant differences between some countries, Greek and United Kingdom voluntary organisations and community groups’ representatives had worked for longer compared to those in the Netherlands or Norway. The average number of years of existence of organisations was 22.4, and significant differences were found between Greece and Spain (p-value = 0.017) and Greece and United Kingdom (p-value = 0.024), as voluntary organisations and community groups were younger in Greece. Other significant differences included the number of volunteers (p-value = 0.003) and paid employees (p-value = 0.001) in the organisations with the lowest mean in the United Kingdom (25.1) and the highest in Spain (1672.1), clearly influenced by the fact that in some countries more representatives from national-larger organisations participated in the study as shown in Table 3 (p-value < 0.001). This was also affecting the reach some organisations have as the reported number of people using the services in a month varied significantly between the participant organisations of the countries (p-valuen< 0.001), having the highest mean in Netherlands (5119.61) and the lowest mean in Norway (469.34) (see Table 2). Further significant differences between the organisations of the participating countries are highlighted in Table 3 in relation to the area where organisations worked and roles of the participants.

**Table 2:** Background information from the survey with representatives of the organisations.

	BULGARIA	GREECE	NETHERLANDS	NORWAY	SPAIN	UK	TOTAL FOR ALL COUNTRIES	P-VALUE* COMPARISON BETWEEN COUNTRIES
**Years working for the organisations in present role**	8.5; 7.92; 6 (3–11)	10.67; 7.65; 9 (5–13)	6.51; 5.83; 5.000 (3–7)	6.81; 7.11; 4 (2–9)	10.31; 9.22; 7 (3–14.75)	10.40; 9.40; 7 (3–14.5)	9.11; 8.25; 7 (3–12)	<0.001* BG-GR = 0.025** GR-NL = 0.002** GR-NO < 0.001** NL-SP = 0.005** NL-UK = 0.005** NO-SP < 0.001** NO-UK < 0.001**
	(N = 147)	(N = 144)	(N = 49)	(N = 145)	(N = 132)	(N = 125)	(N = 742)	
**No years the organisation has existed**	20.1; 24.74; 12 (5.25–20)	18.9; 16.73; 14 (10–20)	24.3; 32.62; 10 (5–30)	21.95; 22.44; 15 (7–30)	25.6; 23.8; 20 (11.25–30)	25.4; 24.9; 19.5 (7–30)	22.4; 23.5; 15 (8–27)	0.002* GR-SP = 0.017** GR-UK = 0.024**
	(N = 144)	(N = 144)	(N = 47)	(N = 140)	(N = 132)	(N = 124)	(N = 731)	
**No volunteers of organisation**	179.6; 1,641.74; 4 (2–13.25)	26.6; 66.52; 7 (3–25)	929.9; 6,072.3; 10 (0–40)	30; 53.9; 7 (0–42.5)	1672.1; 18,089.65; 11.5 (6.25–29)	25.1; 44.32; 10 (3–30)	407.9; 7,788.20; 7 (3–30)	0.003*
	(N = 150)	(N = 144)	(N = 50)	(N = 145)	(N = 132)	(N = 128)	(N = 749)	
**No of paid employees of organisation**	5.2; 39.9; 0 (0–0)	1.60; 4.55; 0 (0–2)	103.3; 326; 6.5 (0–26.25)	6.3; 18,550; 2 (0–5)	81.7; 874.1; 0 (0–1.75)	18.3; 78.4; 1 (0–8)	27; 378.73; 0 (0–8)	0.001*
	(N = 150)	(N = 144)	(N = 50)	(N = 145)	(N = 132)	(N = 128)	(N = 749)	
**No of people using the services of the organisations every month (approx.)**	148.04; 245.15; 60 (30–200)	4781.22; 29,440; 200 (100–500)	5119.61; 17,519.50; 161 (68.75–747)	469.34; 1073.2; 130 (29–400)	2050.88; 15,334.2; 100 (50–400)	3289.93; 24,794.5; 200 (40–867.5)	2575.23; 18,242.15; 140 (45–450)	<0.001*
	(N = 150)	(N = 144)	(N = 46)	(N = 145)	(N = 130)	(N = 121)	(N = 736)	

Mean; SD; Median (P25–P75).

N = number of participants answering this question.

BG (Bulgaria), GR (Greece), NL (Netherlands), NO (Norway), SP (Spain), UK (United Kingdom).

* Kruskall Wallis (non-parametric comparison between countries).

** Fisheŕs Least Significant Difference (LSD) to compare the means of two countries in relation to the number of years the organisations have existed and the number of years the representatives have worked in their role in the organisations.

**Table 3:** Contingency, x^2^ test results from survey with representatives of the organisations (per country).

ACTIVITIES, ROLES & FUNDING DEPENDING ON COUNTRY	BULGARIA	GREECE	NETHERLANDS	NORWAY	SPAIN	UK	TOTAL	P-VALUE
**Area (n** **=** **748)**								<0.001***
Urban deprived	32 (21.3%)	73 (50.7%)	33 (67.3%)	48 (33.1%)	68 (51.5%)	103 (80.5%)	357(47.7%)	
Urban affluent	81 (54%)	21 (14.6%)	14 (28.6%)	48 (33.1%)	45 (34.1%)	25 (19.5%)	234 (31.3%)	
Rural deprived	37 (24.7%)	50 (34.7%)	2 (4.1%)	49 (33.8%)	19 (14.4%)	–a	157 (21%)	
**Best description of role of representative (n** **=** **749)**								<0.001***
Managerial	82 (54.7%)	35 (24.3%)	23 (46%)	112 (77.2%)	72 (54.5%)	69 (53.9%)	393 (52.5%)	
Working with group/participants	37 (24.7%)	66 (45.8%)	14 (28%)	29 (20%)	39 (29.5%)	19 (14.8%)	204 (27.2%)	
Administrative	22 (14.7%)	40 (27.8%)	4 (8%)	3 (2.1%)	13 (9.8%)	13 (10.2%)	95 (12.7%)	
Other	9 (5%)	3 (28.3%)	9 (18%)	1 (0.7%)	8 (6.1%)	27 (21.1%)	57 (7.6%)	
**Level organisation operates at (n** **=** **749)**								<0.001***
Local	96 (64%)	103 (71.5%)	25 (50%)	79 (54.5%)	88 (66.7%)	120 (93.8%)	511 (68.2%)	
Regional	16 (10.7%)	21 (14.6%)	17 (34%)	9 (6.2%)	25 (18.9%)	8 (6.2%)	96 (12.8%)	
National	32 (21.3%)	14 (9.6%)	5 (10%)	5 (3.4%)	11 (8.3%)	0 (0%)	67 (8.9%)	
Local department of a national organisation	6 (4%)	6 (4.2%)	2 (4%)	47 (32.4%)	1 (0.8%)	0 (0%)	62 (8.3%)	
Other	0 (0%)	0 (0%)	1 (2%)	5 (3.4%)	7 (5.3%)	0 (0%)	13 (1.7%)	
**Main activities and services offered by organisation (n** **=** **748)**								<0.001*** Social activities High GDP(39.6%) Low GDP (64%)b = <0.001***
Offering information to public and target groups	31 (20.8%)	50 (34.7%)	12 (24%)	27 (18.6%)	17 (12.9%)	10 (7.8%)	147 (19.7%)	
Offering medical or related help	5 (3.4%)	8 (5.6%)	9 (18%)	16 (11%)	1 (0.8%)	0 (0%)	39 (5.2%)	
Offering practical help	32 (21.5%)	28 (19.4%)	10 (20%)	3 (2.1%)	39 (29.5%)	35 (27.3%)	147 (19.7%)	
Organizing physical activities (exercise)	29 (19.5%)	5 (3.5%)	11 (22%)	42 (29%)	13 (9.8%)	12 (9.4%)	112 (15%)	
Organizing social activities	50 (33.6%)	12 (8.3%)	4 (8%)	40 (27.6%)	48 (36.4%)	28 (21.9%)	182 (24.3%)	
Other^b^	2 (1.3%)	41 (28.5%)	4 (8%)	17 (11.6%)	14 (10.6%)	43 (33.6%)	121 (16.2%)	
**How the organisation is funded (n** **=** **746)** **Charity**								0.010‡
very significant	20 (13.3%)	4 (2.8%)	5 (10.4%)	12 (8.3%)	13 (9.8%)	12 (9.4%)	66 (8.8%)	
significant	27 (18%)	15 (10.4%)	9 (18.8%)	6 (11%)	12 (9.1%)	10 (7.9%)	89 (11.9%)	
N/A	103 (68.7%)	125 (86.8%)	34 (70.8%)	117 (80.7%)	107 (81.1%)	105 (82.7%)	591 (79.2%)	
**How the organisation is funded (n** **=** **747)** **Grants**								<0.001‡
very significant	0 (0%)	32 (22.2%)	16 (32.7%)	88 (60.7%)	51 (38.6%)	28 (22%)	215 (28.8%)	
significant	1 (0.7%)	47 (32.6%)	12 (24.5%)	17 (11.7%)	45 (34.1%)	22 (17.3%)	144 (19.3%)	
N/A	149 (99.3%)	65 (45.1%)	21 (42.9%)	40 (27.6%)	36 (27.3%)	77 (60.6%)	388 (51.9%)	
**How the organisation is funded (n** **=** **745)** **Contributions from members**								<0.001‡
very significant	93 (62%)	67 (46.5%)	11 (22.9%)	60 (41.4%)	36 (27.5%)	44 (34.6%)	311 (41.7%)	
significant	26 (17.3%)	8 (5.6%)	19 (39.6%)	21 (14.5%)	60 (45.8%)	18 (14.2%)	152 (20.4%)	
N/A	31 (20.7%)	69 (47.9%)	18 (37.5%)	64 (44.1%)	35 (26.7%)	65 (21.2%)	282 (37.9%)	
**How the organisation is funded (n** **=** **747)** **Other**^c^								<0.001‡
very significant	16 (10.7%)	70 (53.5%)	25 (51%)	19 (13.1%)	11 (8.3%)	21 (16.5%)	169 (22.6%)	
significant	5 (3.3%)	9 (6.2%)	7 (14.3%)	6 (4.1%)	18 (13.6%)	15 (11.8%)	60 (8%)	
N/A	129 (86%)	58 (40.3%)	17 (34.7%)	120 (82.8%)	103 (78%)	91 (71.7%)	518 (69.3%)	

GDP: Gross domestic product.

*** Contingency, x^2^ test to compare categorical variables of the participating countries in terms of areas from where participants were recruited in each country, roles and level of action and main activities developed.

‡ Contingency, x^2^ test to compare categorical variables of the participating countries in terms of funding.

a Not recorded.

b Literacy, drawing, sewing, food bank, mental health, gastronomic activities.

c Pharmaceutical companies, conference subscriptions, exploitation of assessments, advertisement.

Table 3, Table 4 present significant differences between countries and areas in relation to roles (p-value < 0.001), activities (p-value < 0.001) and funding sources (p-value < 0.001).

**Table 4:** Contingency, x^2^ test results from survey with representatives of the organisations (per area).

ACTIVITIES & FUNDING DEPENDING ON AREA	URBAN DEPRIVED	URBAN AFFLUENT	RURAL DEPRIVED	TOTAL	P-VALUE
**Main activities and services offered by organisation (n** **=** **747)**					0.007‡‡
Offering information to public and target groups	75 (21%)	41 (17.5%)	30 (19.2%)	146 (19.5%)	
Offering practical help	76 (21.3%)	55 (23.5%)	16 (10.3%)	147 (19.7%)	
Offering medical or related help	17 (4.8%)	15 (6.4%)	7 (4.5%)	39 (5.2%)	
Organizing physical activities (exercise)	45 (12.6%)	44 (18.8%)	23 (14.7%)	112 (15%)	
Organizing social activities	80 (22.4%)	52 (22.2%)	50 (32.1%)	182 (24.4%)	
Other	64 (17.9%)	27 (11.5%)	30 (19.2%)	121 (16.2%)	
**How the organisation is funded (n** **=** **746)** **Charity**					
very significant	35 (9.8%)	16 (6.9%)	15 (9.6%)	66 (8.8%)	
significant	41 (11.5%)	30 (12.9%)	18 (11.5%)	89 (11.9%)	
N/A	280 (78.7%)	187 (80.3%)	124 (79%)	591 (79.2%)	
**How the organisation is funded (n = 746)** **Grants**					<0.001‡‡
very significant	117 (32.9%)	61 (26.2%)	36 (22.9%)	214 (28.7%)	
significant	80 (22.5%)	27 (11.6%)	37 (24.5%)	144 (19.3%)	
N/A	159 (44.7%)	145 (62.2%)	84 (53.5%)	388 (52%)	
**How the organisation is funded (n** **=** **746)** **Contributions from members**					
very significant	139 (39.2%)	110 (47.2%)	62 (39.5%)	311 (41.7%)	
significant	74 (20.8%)	38 (16.3%)	40 (25.5%)	152 (20.4%)	
N/A	142 (40%)	85 (36.5%)	55 (35%)	282 (37.9%)	
**How the organisation is funded (n** **=** **747)** **Other**^a^					<0.001‡‡
very significant	82 (23%)	41 (17.6%)	46 (29.3%)	169 (22.7%)	
significant	42 (11.8%)	11 (4.7%)	6 (3.8%)	59 (7.9%)	
N/A	232 (65.2%)	181 (77.7%)	105 (66.9%)	518 (69.4%)	

‡‡ Contingency, x^2^ test to compare categorical variables of the three geographical areas in terms of activities developed by the organisations in each area and funding.

a Pharmaceutical companies, conference subscriptions, exploitation of assessments, advertisement.

Greece had a higher number of representatives (n = 66) who directly offered practical help to the members of the organisations compared to other countries in which representatives participating in the study mainly developed managerial roles (n = 112 in Norway and n = 82 in Bulgaria for example).

Regarding activities developed by the organisations, “offering medical help” was the least frequent activity (5.2%). “Offering practical help” related activities varied across countries (p-value < 0.001) and areas (p-value = 0.007), being less frequent in Greece (19.4%) and Norway (2.1%) and in rural deprived areas in all the participating countries (39/747 representatives). Furthermore, there were significant differences (p-value < 0.001) when comparing countries with lower (Bulgaria, Greece and Spain) and higher (Norway, Netherlands and United Kingdom) gross domestic product as in non-affluent countries organisations tend to promote social activities twice as often (64% vs 39.6%). Likewise, in proportion organisations in rural deprived areas (50/166) focused more on social activities than those in urban deprived (80/357) and urban affluent (52/234) areas.

Finally, there were significant differences in the funding sources, depending on the country (p-value < 0.001). Grants and contributions from members were the most important sources of money for the organisations (significant and very significant in 48.1% and 62.1% of the cases respectively).

Significant differences were also found according to the area and source of funding (p-value < 0.001) for grants (very important in 117/356 cases in urban deprived compared to 36/157 cases in rural deprived).

Considering the research questions of this paper, the results from the survey in this paper provided very useful information about the organisations, which helps contextualize the findings from the interviews below. Furthermore, this part of the study was essential to identify issues mainly related to the type of activities, roles and sources of funding, which need further in-depth explanation.

### 3.2 Themes emerging from the interviews

A total of 4 themes and 15 subthemes finally emerged from the analysis to answer the research questions of this paper (see Box 1). In line with the quantitative results above, the findings from the interviews provided rich details and contributed to elaborate more on some of the differences and issues identified in the survey.

Box 1 **Themes and subthemes emerging from the coding process.**

**Table:** 

Theme 1. Better reach than formal services: networking and referral Subthemes: Reach capacity, Mediation between macro and micro, Mediation between macro and meso, Connections with technological companies
Theme 2. Meeting basic practical needs: filling the gaps left by formal services Subthemes: Practical basic needs management and prevention of complications, Information and education
Theme 3. A complement to formal services: Humanizing care to increase wellbeing Subthemes: Quality of life for everyone, Normalisation and socialisation, Financial crisis, Safety and wellbeing, Organisations filling the family gap
Theme 4. Sharing more than an illness: mediators between services and communities Subthemes: Illness experience, Illness management, Social inclusion-you are not alone-, Culture and beliefs

Alt-text: Box 1

#### 3.2.1 Theme 1. Better reach than formal services: networking and referral

Reach and engagement capacity of the voluntary organisations and community groups was highlighted in most interviews and defined the relationships between all the sectors (macro, meso, micro). Users benefited from engaging with voluntary organisations and community groups as this provided access to other services and a smoother transition between levels of care.

*“Some years ago we organized a group with diabetic patients because in the primary health centre they were interested in it (group) and knew that within the Roma community in X district there were many diabetic patients we had access to.” (Spain 2-Healthy lifestyle)*

*“My members discuss it as falling through the black hole when you come out of hospital, you seem to get so much support when you're in there but then when you come home it's just what now, you know, and people feel very isolated and left to their own devices and… I mean they might not need us at that point and they're certainly not looking to do social activities… …but that's where the peer support service comes into its own at that point because obviously I think when people are in hospital they don't really know what they want when they get home.” (United Kingdom 40-Illness related)*

Moreover, interviewees stated that because they had better reach than formal health and social services they were in a better position to identify and look after the social, cultural and mental health daily needs of people.

“*… when you work with certain types of populations, and you need people who belong to the community… to help… the professional deals with health issues and the volunteer watches over the cultural keys and translates the information for families… …volunteers cańt cross that line…”(Spain 6-Healthy lifestyle)*

This was especially relevant in rural or deprived communities, which were more difficult to recruit, work with or promote healthy behaviours in. Some organisations stated that because of their ability to reach out and include people with diabetes they helped professionals refer patients to the most relevant local organisations. This involved work on the part of the voluntary organisation and community groups to publicize their services and ensure their presence in key health committees and meetings. In the Netherlands general practitioners and hospitals were the main sources of referral into voluntary organisations and community groups.

*“...We have a number of different physical activity and weight management contracts with the Primary Care Trust… working in partnership with local health professionals…they refer patients to us which we then signpost into lots of different types of physical activity…” (United Kingdom 22-Healthy lifestyle)*

“*Through referral; by GP’s, hospitals, informal care. Everyone has* access to us” (Netherlands 46-Illness related).

Nevertheless, in some countries like Norway referral was not happening mainly as a result of the reluctance of some professionals to use lay community resources.

“*For quite some time now it has been up to the GP’s and how they think about it. If they are aware that there are Learning and mastery centres holding courses for patients with diabetes type 2 or to…like a walking group in the healthy life centrals and then if they then refer patients to these… … In a way it’s the GP who holds the key to the other measures. So if you have a…. hmmm…. a conscientious GP and then it can work really well*.” (*Norway 106-Illness related*)

#### 3.2.2 Theme 2. Meeting basic practical needs: filling the gaps left by formal services

Participants stated that they had an important role in giving practical advice to users of the organisations, and acting as a repository of skills.

Promoting safe management through addressing basic practical needs was important to decrease or delay dependence and the provision of some education/information was also essential (diet, foot care and physical activity, means of transport, legal and practical advice, or employment).

*“Foot care… this is important and we need to increase awareness of the importance of foot care in many people… especially in some small villages with a small and very elderly population… we sometimes ask the podiatrist to visit them… if they are in bed and cańt move…” (Spain 48-People’s and patients’ rights)*

However, the financial difficulties of some users were perceived as a barrier to following the advice.

*“We take glucose and blood pressure readings daily. We speak to them about right nutrition. There are, however, individuals who cannot even afford to buy their medicines.” (Greece 32-Wellbeing)*

In Spain, Netherlands and the UK some organisations expressed general rather than specific concerns to increase the self-management skills of members. More concretely, in the Netherlands special efforts were made to increase self-reliance by creating specific environments or a culture in which patients could daily function or gain further awareness of the importance of self-managing.

However, in Greece and Bulgaria organisations were involved in improving the uptake of advice provided through self-management educational programmes, in some cases very pragmatically through reducing the price of medicines and insulin pumps and in others by signposting sources of good treatment.

”*We, NGOs [non-governmental organisations] in fact do what the state’s work is, instead of doing concrete tasks… we have to do the state’s work − to inform the patients, to give advice on where and when they should look for treatment, to work for prevention, to establish equal treatment for patients with different diseases…”. (Bulgaria 17-People’s and patients’ rights)*

As a result of developing these activities voluntary organisations and community groups sometimes experienced negative relationships with formal services, this centred around the notion of a “substitute role”, which often emerged in the interviews. This was clearly influenced by the financial crisis experienced in Europe, which resulted in fewer resources in formal services, and organisations saw themselves rescuing deficient systems as on many occasions they were considered a cheap resource.

According to interviewees these negative feelings of “filling the administration gaps” or “being used” seemed to emerge from unequal relationships, blurred roles, the lack of acknowledgement from health professionals and administrators of the voluntary organisations and community groups’ work and the poor synchronization in self-management or other health initiatives of both sectors.

*“I guess that both the administration and the third sector want the same thing, the opposite would be absurd but when it comes to implementing initiatives it seems that formal services have a different objective. We as an organisation want more implication of formal services and to be able to have further resources, their resources…” (Spain 16-Illness related)*

#### 3.2.3 Theme 3. A complement to formal services: humanising care to increase wellbeing

Several participants stated that they perceived diabetes self-management more holistically and felt their role in self-management initiatives was complementary rather than substitute because they could support and focus on the *self* component of self-management. Voluntary organisations and community groups placed a special emphasis on individualizing and humanising services and not exclusively on technical care through time investment and high levels of engagement with the target population.

“*In the Netherlands wellbeing organisations traditionally have a complementary function in relation to the health system. Whereas professional health care focuses on physical well-being, wellbeing organisations predominantly have a focus on psychosocial wellbeing. However, the scope of wellbeing organisations is becoming more important as the government is reducing its tasks. For example, elderly are supposed to live in their own house longer instead of going to a house for elderly. For this group, wellbeing organisations now have an extra task to create the possibility for these people to maintain an active and social life. We create the possibility for people to participate in activities and improve their wellbeing*” *(Netherlands 9-Wellbeing)*

An increased perception of wellbeing was achieved by building supportive relationships with users, fighting prejudices about diabetes and offering opportunities. Voluntary organisations and community groups considered themselves a gateway for re-engagement with society and fostering increased social cohesion and social capital.

*“Before the founding of our society there was a lot of prejudice on the subject of diabetes and towards individuals with diabetes. With the actions of the society this was overcome to a great extent; people started to talk about their problem, to accept it, to admit it.” (Greece 1-Illness related)*

Furthermore, when it came to describing more vulnerable individuals with very limited personal network support, safety and mental health became paramount to ensure wellbeing because voluntary organisations and community groups were seen as a place where illness was not the straightforward focus. Therefore, concepts like proximity and intentional presence were viewed as vital for the success of health programmes and the management of long term needs.

*“Being there to accompany the person or provide respite at home or in clinical centres, nursing homes… we can tell that people feel lonely… this is a new illness we are identifying* ‘*loneliness*’*… … so being able to follow up these people for voluntary organisations like ours is important”. (Spain 32-*People’s* and patients’ rights)*

*“…you will find that people will talk about things…in a more friendly environment than they will in a, in a professional one… they’re more inclined to open up…” (United Kingdom 9-Community group)*

To make this ideal complementary role more effective in health and self-management initiatives, relationships with formal services needed to be healthy and based on collaboration, commissioning and consultation. However, executive/hierarchical collaboration could jeopardize complementary relationships when voluntary organisations and community groups were implementing or managing health programmes in some communities, when they were driven by policy agendas or supervised by formal services, or when voluntary organisations and community groups did not feel completely involved in policy making in relation to diabetes regional or national management. Several organisations only felt part of top-down relationships, which were not generally mutually beneficial.

”*… Here, we are fighting with the state bureaucracy⋯Instead of dealing with the patients’ social life, we fight to defend the interests of the ill people…” (Bulgaria 23-*People’s* and patients’ rights)*

Finally, in spite of the important role of voluntary organisations and community groups in humanising care, some organisations depended on the provision of limited funding for which they had to compete and then manage and account for in a bureaucratic process. This has resulted in “professionalizing” the third sector and possibly decreasing the number of self-contained altruist organisations.

*“Obviously, as you get bigger, the amount of money you have to bring in is bigger so, you know, it́s harder. I think that, the minute you mention art type things, people think, oh, theýre sort of fluffy… and, you know, so people who would be in commissioning would not normally want to give you some money for things” (United Kingdom 38-Wellbeing)*

#### 3.2.4 Theme 4. Sharing more than an illness: mediators between services and communities

The voluntary organisations and community groups representatives played a mediating role between macro and micro levels, helping professionals approach communities to spread/implement health programmes.

*“Professionals normally work in a consultation or an office or health centre… that sets up some distance, doesńt it*? *with the communities and target groups, it is good but sometimes it makes work more difficult… then the closeness to the community that a native volunteer can offer and working with them are essential.” (Spain 2-Healthy lifestyle)*

People with diabetes or other long-term conditions could join voluntary organisations and community groups to gain access to services, and to feel supported in the self-management learning process. Voluntary organisations and community groups were able to offer the sort of support that was not found in formal services to reinforce the advice and skills gained through self-management programmes. A good example of this was the peer support received from people who shared their experience in illness and management, in a social non-clinical setting. This allowed positive distraction from everyday health concerns, increased social inclusion, enabling a focus on healthy life rather than diseases.

“*People meet locally. It’s based on groups – not diagnosis. But what happens is that processes start by meeting someone that said something that made sense related to managing everyday life. It’s close to where you live and the courses we give are really cheap” (Norway 107-Healthy lifestyle)*

The mediating role of the voluntary organisations and community groups was also evident when, as stated above, organisations had to negotiate healthy behaviours with certain communities on behalf of health professionals especially with those communities where formal services had a poor reputation. Voluntary organisations and community groups provided translation and transmission of messages from formal services.

*“We like many other patients and families who live with a long term condition see that the system has many holes… generally speaking… poor information, services etc… we as a group cover some areas in which we complement the formal services… the Administration talks about self-management and only want to reduce the number of people going to the consultations… but for us self-management is a responsibility… … we try to deepen in or improve what the professionals tell patients and families…” (Spain 4-Illness related)*

“*We help with the social consultations and assist patients by going with them to meet the Administration…they need us because we know how to protect them….we try to be versatile ….and opposite to bureaucracy…” (Bulgaria 21-*People’s* and patients’ rights)*

An emerging phenomenon of “socializing health” was evident in descriptions of anti isolation strategies, offering a sense of belonging and fitting in, sharing hobbies and skills, and building long-standing relationships. Organisations seemed to play an essential advocate role to building resilience and solidarity, especially in out of reach or deprived communities as it is illustrated in the next Bulgaria quote:

”*When we had to prepare ”martenichki” [thread jewellery every Bulgarian gets from friends and family on the 1 of March] the ”professionals” among us educated the rest how to do this… Men also took part, and even found it amusing⋯Younger pensioners have less time, because most of them try to work…they would have felt better, their life would had been more interesting⋯Especially those that got diabetes recently, we could teach them about diets…” (Bulgaria 7-Wellbeing)*

## 4 Conclusions

This study illuminates how the ethos and work of the voluntary organisations and community groups could build constellations of working relationships which implicated elements of the micro and macro societal levels for diabetes self-management and in turn influenced the type of interventions and barriers that these relationships and roles involved.

**Fig. 1:**
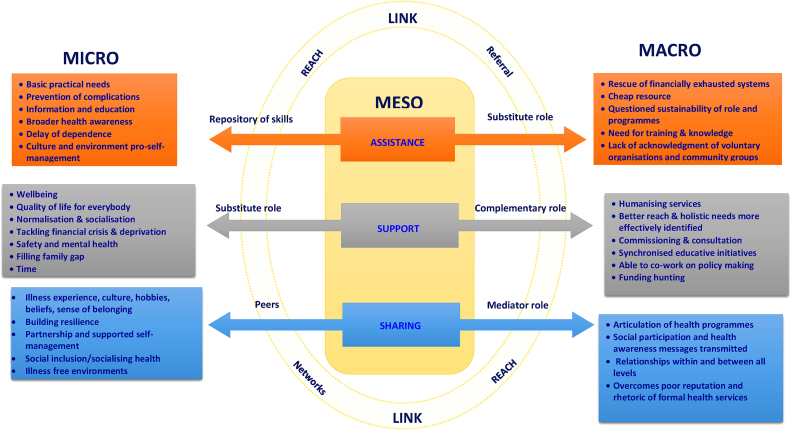
Working relationships and interventions for diabetes self-management and health programmes in Europe: principles of assistance, support, sharing and link.

In considering the three research questions of this paper, Fig. 1 shows how the principles of assistance, support, sharing and link found in the literature (Portillo et al., 2015) were operationalized according to the empirical data obtained in this current FP7 study in Europe. More concretely, Fig. 1 shows:

1. An outline of the key activities and working relationships of the organisations in relation to professionals and users levels in relation to each principle (see boxes under macro and micro levels).
2. The organisations’ roles when developing these activities and building working relationships.
3. In the middle circle of the figure some key linking elements of the organisations when working with users (micro) and professionals (macro) such as their reach and network capacity, and the need for the referral of cases.

Our findings suggest that although the principles of assistance, support, sharing and link (Portillo et al., 2015) which informed the original research questions were clearly found across countries, there is little unanimity across Europe in the definition of diabetes self-management interventions developed or promoted by voluntary organisations and community groups. More concretely, in the UK, Norway and the Netherlands voluntary organisations and community groups provided a broader approach to support health and wellbeing with no clear focus on self-management. On the other hand, in Bulgaria, Greece and Spain findings suggested that in spite of being new in the development and implementation of self-management health policies or because of that, voluntary organisations and community groups focused on the promotion of self-management.

The principle of *assistance* was a key concept emerging in the study in both the survey and the interviews and referred to the management of diet, physical activity or medication. A clear concern emerging was that volunteers seemed to take over medical responsibilities (especially in countries experiencing financial health cuts) and therefore, further research on the implications this may have for diabetes care is needed. The quantitative results of the study underscored the range of capacity of the organisations to support their members based on the number of paid workers and volunteers and also the financial support received, varying in countries and geographical areas. This clearly could justify some of the issues revealed in the interviews, which the survey could not unfold, like the financial limitations some users experienced to follow the practical help given, how the organisations operated to promote healthy behaviours in the target population or the feelings emerging in the adoption of these roles and the long term sustainability of their interventions, considering their resources. Furthermore, although the survey showed that offering practical help was important for organisations in most participant countries, interviews also showed that the understanding or focus of this practical help slightly varied across countries as it was influenced by the organisations ethos or the priorities of the target population they worked with.

The principle of *support*, involving emotional and social level support, resonated with the shifting perspective model of long-term conditions where it is recognized that individuals with long-term conditions can shift focus between foregrounding wellness or illness in different periods (Morris et al., 2011, Tricco et al., 2012, World Health Organisation, 2016). In this sense, the humanization of services emerges as another strong self-management initiative in line with person centred care, which is the backbone of self-management support but a great challenge for self-management policy makers and health professionals (Kennedy et al., 2014, Powers et al., 2015, Pumar-Méndez et al., 2016).

The principle of *sharing* was a defining feature of voluntary organisations and community groups because organisations users and volunteers shared gender, culture, illness, and life experiences and this resulted in greater feelings of social inclusion, safety and wellbeing, essential for the success and operationalization of self-management (Bossy et al., 2016, Poortinga, 2012).

These two principles clearly evident in both the survey and the interviews data indicated that the sense of community and socialisation were strong features of the activities and messages promoted by the organisations and could also foster the sustainability of the organisations-based on the mobilisation of financial support and collective beliefs.

Finally, the principle of *linking* encapsulated all the efforts that voluntary organisations and community groups made to strengthen or ensure the access of users to community resources and health systems. This completes the principles (Fig. 1) to guarantee the success of several self-management or global health programmes and improve the poor reach into deprived or isolated communities, which constitutes a weakness in self-management programmes (Dixon et al., 2011, Kennedy et al., 2013, Wensing et al., 2014). In the survey the figures provided by the organisations about the number of people using their services every month were approximate. In many organisations there was no official register of the users every month and data did not specify if users were different every time or recurrent cases. This does not discredit the organisations’ reach capacity but requires a cautious interpretation of these data. Notwithstanding, the qualitative and quantitative findings of the study were consistent and associations between the organisations’ collaboration with health professionals and their reach could be drawn from the data. In particular, countries like Netherlands which seemed to have greater referral of cases from health professionals, also reported the highest number of people using their services, which contrasted with the situation in Norway where participant organisations stated poorer levels of referrals from professional sources.

Most of the representatives from the participant organisations conceptualized broader forms of diabetes support that extended beyond those adopted and promulgated in health services. Bottom up diabetes self-management initiatives are from our data seemingly emerging strongly pointing a clear shift from a specific illness focus to holistic complexity, and aspirations for social cohesion, a sense of community and wellbeing (Nolte et al., 2014). This had particular resonance in deprived communities and in countries with lower gross domestic product in which special emphasis is needed to empower more disadvantaged people, and in the planning and implementation of more realistic self-management programmes, which should contemplate patients’ culture and normality (Wilkinson et al., 2014). Further research on this is needed to determine if this is a consequence of the economic crisis or if “socializing health” is the key for the success of diabetes self-management rather than education on symptom/treatment management (Collinsworth et al., 2013, Lorig et al., 2010). As shown in the quantitative and qualitative data, the third sector has gained importance in health promotion throughout Europe due to the financial crisis. However, these organisations, relying on funding coming from grants and memberships, are experiencing financial cuts, which detract from the key complementary services they are providing. In this regard, European and International policy thinking should reflect on reality, plan strategies to overcome boundaries between structures and sectors, and broaden the perspectives of healthcare teams to include more tangible social participation in plans concerning self-management policies, implementation of programmes, intersectoral relationships, role definition and funding distribution not only for diabetes but also for other long-term conditions.

As for the third research question, this paper has shed light on the types of working relationships between voluntary organisations and community groups, health systems and users that are emerging in the implementation of self-management programmes in Europe.

The fact that relationships between levels of actions were not always as expected or anticipated (lack of acknowledgement, need for more synchronization, feelings of being used) resulted in the need to plan certain activities (more practical and educational help, mediation between communities and formal services, tackling deprivation) to promote healthy behaviours, operationalize some health programmes and fill some action-planning gaps. Even if in this study it remained unclear what emerged first (roles/activities or relationships between sectors), peer intersectoral collaboration is required to cope with diabetes holistic consequences and create integrated health-supporting environments (World Health Organisation Regional Office for Europe, 2011).

This study presented a mixed method design, counterbalancing the limitations of different methods and increasing the validity and reliability of the study (Creswell and Plano Clark, 2011, Jick, 1979) and results obtained from the qualitative and quantitative sets of data were congruent and complementary (Elissen et al., 2013), providing a rich understanding of the working relationships, roles and interventions of the voluntary sector in diabetes self-management and how the principles of assistance, support, sharing and link found previously are operationalized from a more diverse set of perspectives and examples. Results from the quantitative comparison between countries should be interpreted with caution because the survey with representatives of organisations was observational, and statistical generalisation was not sought in this study. Furthermore, results did not show causal relationships and cannot be considered representative of all the voluntary organisations and groups in all the participating countries. However, one of the strengths of this paper is its multicultural and multinational nature, showing a wide perspective from participants representing organisations from varied settings in the participating countries (recruitment took place at national and local levels, and in urban affluent, urban deprived and rural deprived areas in most participating countries).

The multicultural and multinational nature of our data could also bring limitations in terms of the validity of the qualitative data because some language specific meanings could have been lost during the translation process, and the reporting of the findings of the qualitative analysis in each country could not be identical as only the confirmation of the whole analysis process took place between Spain and UK. However, as stated above measures were implemented to ensure rigour and minimize discrepancies (Temple and Young, 2004) and groups discussions were essential to build concepts from the findings, which were beyond mere language translation.

Lastly, the mixed method approach has also provided rich contextual information, identified key issues emerging about the involvement of European voluntary organisations and community groups in diabetes self-management and helped understand the rationale behind this. Also this study has shown an original knowledge leap in relation to diabetes self-management and strongly illustrated a new health care movement in Europe.

## Conflict of interest

The authors declare no conflict of interest.

## Funding

This study has been funded by the 7th Framework Programme of the European Commission (grant agreement number 279081).

## Author contributions

M.C.P. researched and analysed data and wrote the manuscript. A.K. researched and analysed data and reviewed and edited the manuscript. E.T. researched and analysed data and reviewed and edited the manuscript. E.R. researched and analysed the data and edited the manuscript. M.W. designed and coordinated the international survey study and edited the manuscript. C.F. researched and analysed the data and edited the manuscript. C.L. was involved in all stages of the project and edited the manuscript. I.V. was involved in all stages of the study and edited the manuscript. V.G. analysed data and provided statistical advice and edited the manuscript. A.R. was the EUWISE coordinator and was involved in all stages of the study and edited the manuscript.
